# Complete genome sequence of three atypical diarrheagenic *Escherichia coli* O166:H15 strains from foodborne outbreaks

**DOI:** 10.1128/mra.00746-25

**Published:** 2025-10-09

**Authors:** Akiko Kubomura, Kenichi Lee, Sunao Iyoda, Yukihiro Akeda

**Affiliations:** 1 Department of Bacteriology I, National Institute of Infectious Diseases, Japan Institute for Health Security 739298 , Shinjuku, Japan; Loyola University Chicago, Chicago, Illinois, USA

**Keywords:** *Escherichia coli*, diarrhea, foodborne

## Abstract

*Escherichia coli* O166:H15 strains have been repeatedly isolated from foodborne outbreaks in Japan. These strains lack the major virulence factors typically associated with diarrheagenic *E. coli*. To characterize the genomic features of these atypical strains, we present the complete genome sequences of three *E. coli* O166:H15 isolates from foodborne outbreaks.

## ANNOUNCEMENT

Diarrheagenic *Escherichia coli* (DEC) is classified into five major pathotypes, including Shiga toxin-producing *E. coli* and enterotoxigenic *E. coli* (1). Each pathotype is characterized by specific virulence genes, such as *eae* (encoding intimin) and *elt* (encoding heat-labile toxin). However, in Japan, *E. coli* O166:H15 strains that lack these known virulence genes have been associated with several foodborne outbreaks over the past 30 years (2, 3). To investigate their genomic features and potential pathogenicity, we determined the complete genome sequences of three *E. coli* O166:H15 isolates.

These isolates were obtained from three independent foodborne outbreaks (Table 1). In all cases, identical strains were isolated from multiple patients, and no other pathogens were detected. On the basis of these findings, *E. coli* O166:H15 was determined to be the causative agent. The strains were recovered from clinical samples by local public health institutes and subsequently transferred to the National Institute of Infectious Diseases. Bacterial cultures were grown overnight in buffered peptone water (Nissui Pharmaceutical, Japan) at 37°C, and genomic DNA was extracted using the MagMax DNA Multi-Sample Ultra 2.0 Kit with the KingFisher Duo Prime system (Thermo Fisher Scientific, USA). PCR screening confirmed that none of the isolates possessed known DEC marker genes, including *stx1/2*, *elt*, *estA1/2*, *invE*, *eae*, *aggR*, and *afaD* (Table S1. https://doi.org/10.5281/zenodo.17090503). The subsequent genome analysis using VirulenceFinder (https://cge.food.dtu.dk/services/VirulenceFinder/) also did not detect any major virulence factors, including adhesins, invasion factors, or toxins. Among the three isolates, more than 200 core genome SNPs were identified.

**TABLE 1:** Genome metrics and accession numbers of strains

Isolate name	Isolation year	No. of patients identified with the strain	Location	Chromosome size (bp)(accession no.)	Plasmid size (bp) (accession no.)	No. of CDS	No. of rRNAs	No. of tRNAs	G + C content (%)	Short read			Long read			
										No. of reads	Coverage	DRA accession no.	No. of reads	Coverage	N50	DRA accession no.
JNE070796	2006	147	Kumamoto	5,051,314 (AP041805)	138,061 (AP041806), 91,511 (AP041807), 5,831 (AP041808)	5,108	22	92	51	2,116,954	108.2	DRR707029	16,016	46.0	23,721	DRR707030
JNE21-003	2021	181	Saitama	5,079,930 (AP041809)	1,399,961 (AP041810), 84,642 (AP041811), 3,345 (AP041812), 1,945 (AP041813)	4,943	22	91	51	2,057,706	105.4	DRR707031	47,985	152.1	25,040	DRR707032
JNE21-009	2016	28	Hyogo	5,144,898 (AP041814)	188,563 (AP041815), 163,363 (AP041816), 2,012 (AP041817)	5,418	22	96	51	1,157,728	52.4	DRR707033	47,676	139.7	23,745	DRR707034

For short-read sequencing, genomic DNA libraries were prepared using the QIAseq FX DNA Library Kit (Qiagen, Germany), followed by paired-end sequencing (2 × 300 bp) on the MiSeq platform (Illumina, USA). For long-read sequencing, libraries were prepared using the Rapid Barcoding Kit V14 (SQK-RBK114.24, Oxford Nanopore Technologies, UK) and sequenced on a MinION Mk1B using an R10.4.1 flow cell over a 72-h run. Basecalling was performed using Dorado v0.9.5 with a super-accurate model (dna_r10.4.1_e8.2_400bps_sup@v5.0.0). Quality of the reads was assessed by SeqKit v2.10.0 (4), QUAST v5.2.0 (5), and CheckM v1.2.4 (6). Contamination and completeness calculated by CheckM were below 2% and above 99%, respectively, in all the isolates. To obtain complete genomes, hybrid assemblies were generated using Hybracter v0.11.0 (7) (JNE070796 and JNE21-003) or Autocycler v0.4.0 (8) (JNE21-009). Genomes assembled with Autocycler were subjected to short-read polishing by Polypolish v0.5.0 (9) and PyPolca v0.3.1 (10, 11) and long-read polishing by Medaka v0.2.0 (Oxford Nanopore). Genome annotation was carried out using the NCBI Prokaryotic Genome Annotation Pipeline (PGAP) v2025-05-06 (12), followed by manual curation. All analyses were performed using the default parameter settings.

Since we only used completely anonymized patient information, approval from our institutional ethics committee was not required.

## Data Availability

The genome information, including accession numbers, is summarized in Table 1. The genome sequences have been deposited in DDBJ/ENA/GenBank under BioProject number PRJDB35536.
